# Dietary Fatty Acids Modulate Gut Microbiota-Derived Trimethylamine-N-Oxide: Potential Mechanisms and Future Perspective

**DOI:** 10.3390/nu17233787

**Published:** 2025-12-03

**Authors:** Ece Kilic, Pervin Basaran

**Affiliations:** Department of Food Engineering, Faculty of Chemical and Metallurgical Engineering, Istanbul Technical University, Maslak, 34469 Istanbul, Turkey; basaranakocakp@itu.edu.tr

**Keywords:** fatty acids, cardiovascular disease, biomarker, trimethylamine N-oxide

## Abstract

High-fat diets are known to contribute to metabolic disorders such as obesity, diabetes, and cardiovascular diseases, partly through alterations in gut microbiota composition. However, the impact of dietary fat on gut microbiota depends on fat composition, with both the degree of saturation and chain length of fatty acids playing essential roles in modulating microbial populations. Saturated long-chain fatty acids have been shown to promote the production of trimethylamine (TMA), a precursor of trimethylamine N-oxide (TMAO), an emerging gut microbiota-derived biomarker associated with cardiovascular disease. These effects occur through multiple mechanisms, including increased colonic oxygen levels and taurine-conjugated bile acids, which promote pathways that favor TMA-producing bacteria. In contrast, short-chain fatty acids (SCFAs) and omega-3 polyunsaturated fatty acids exert beneficial effects by altering pH and supporting SCFA-producing bacteria, thereby reducing levels of TMA-producing bacteria. Given the influence of gut microbial communities and their metabolites on the onset of metabolic disorders, dietary strategies that modulate the microbiota and its metabolic products through optimized fatty acid composition represent promising therapeutic approaches for preventing conditions such as cardiovascular disease.

## 1. Introduction

**Figure 1:**
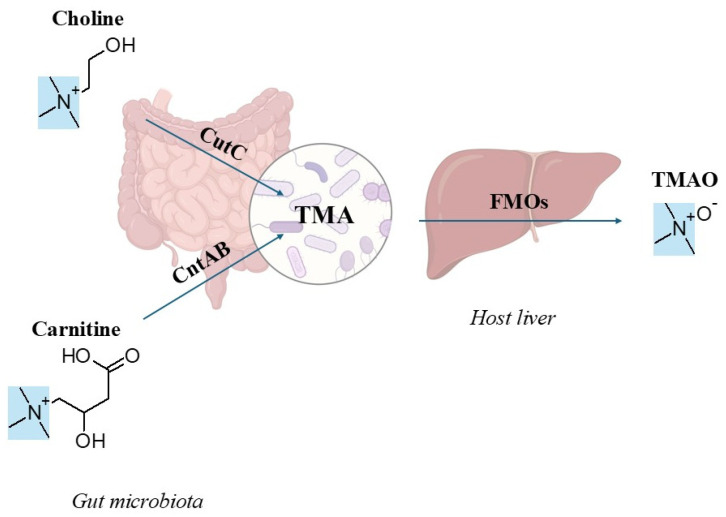
Trimethylamine (TMA), a tertiary amine (highlighted in blue), is a common structural component of nearly all trimethylamine-N-oxide (TMAO) precursors. Excess dietary precursors, primarily choline and carnitine, are metabolized into TMA by gut microbial enzymes (CutC: choline-TMA lyase; CntAB: carnitine monooxygenase). TMA undergoes oxidation in the host liver by flavin monooxygenases (FMOs) to form TMAO. (The chemical structures were drawn with ChemSketch, Version 14.01; Advanced Chemistry Development, Inc. (ACD/Labs): Toronto, ON, Canada, 2012.).

Trimethylamine-N-oxide (TMAO), gut microbiota-derived metabolite, is associated with a higher risk of cardiovascular disease (CVD) and may serve as a biomarker for heart failure [1]. However, the underlying mechanisms remain complex and poorly understood [2], partly because circulating TMAO levels are influenced by diet, the gut microbiome composition, and host metabolism. Studies in humans, animals, and cell models demonstrate that elevated plasma TMAO levels adversely affects multiple organs, particularly the heart, liver, and kidneys [2,3,4,5,6,7]. As the kidneys primarily eliminate TMAO from the body, impaired renal function may both intensify its harmful effects [8], and elevate TMAO levels, complicating causal interpretations [2]. TMAO production is a metaorganismal process in which gut bacteria convert dietary precursors into trimethylamine (TMA), which is then oxidized to TMAO in the host liver, facilitated by flavin monooxygenase (FMO) enzymes (Figure 1) [9,10,11]. Choline and carnitine are the major dietary precursors for gut microbiota-dependent synthesis of TMAO [12]. Phosphatidylcholine, the primary dietary source of choline, is plentiful in foods of both plant and animal origin, while carnitine is mainly present in red meat [4,12]. It is well established that gut microbiota is essential for converting these metabolites into TMA, as gnotobiotic mice (which lack microbiota) do not produce TMA. Additionally, treating conventional mice with antibiotics significantly reduced TMA formation. When gnotobiotic mice are colonized with choline-metabolizing bacteria, cecal TMA production increases [13]. These TMA-producing bacteria harbor genes encoding enzymes essential for TMA synthesis, including choline-TMA lyase (CutC), carnitine monooxygenase (CntAB), glycine betaine reductase (GrdH), and TMAO reductases (collectively TorA) [14,15,16,17]. Recently, the YeaW/X enzyme complex (dioxygenase and oxidoreductase) was proposed as a third major pathway that directly converts γ-butyrobetaine (γBB) to TMA [18].

Gene clusters involved in TMA production within the gut microbiota are generally found in both obligate anaerobes from the Clostridia class (Firmicutes) and facultative anaerobes from the *Enterobacteriaceae* family (Proteobacteria) [17,19]. In vitro studies using bacterial isolates from the human intestinal tract revealed that certain strains produced TMA only when choline was provided, as demonstrated by stable isotope tracer experiments [20]. In contrast, carnitine did not serve as a TMA precursor under the same experimental conditions. Carnitine conversion to TMA requires oxygen-dependent enzymes, which were initially discovered in *Acinetobacter* species and later identified in other gut bacteria. However, given the anaerobic nature of the intestinal environment, the activity of these enzymes is likely constrained [21]. Alternatively, a plausible anaerobic pathway for TMA production from carnitine has been proposed. In this pathway, carnitine is first converted to γBB, which is then converted to TMA. This multistep process appears to require cooperation among different gut commensals, with the obligate anaerobe *Emergencia timonensis* identified as a key bacterium capable of converting γBB into TMA. Thus, *E. timonensis* may play a crucial role in TMA production following carnitine consumption in humans [22].

The predominantly anaerobic gut environment favors anaerobic TMA-producing pathways, particularly those mediated by TorA, CutC, and GrdH. Among these, TorA was the most frequently observed, suggesting an active cycle between TMAO and TMA in the gut environment. However, since dietary TMAO primarily originates from marine fish, choline remains the main dietary precursor of TMA. Therefore, the CutC pathway is the primary route of TMA production in the gut. GrdH-like pathways are limited because the energy-demanding synthesis of glycine betaine makes its breakdown metabolically unfavorable in the gut [15].

Although anaerobic choline metabolism is known to play a significant role in CVD, the identity of the key gut microbes involved remained uncertain until the recent discovery of a gene cluster in *Desulfovibrio desulfuricans* and *Desulfovibrio alaskensis* that mediates this pathway [23]. This finding aligns with earlier work showing that CutC was first identified in the anaerobic sulfate-reducing bacterium *D. desulfuricans* [24]. Diet further shapes this metabolic axis [25]. Western dietary patterns, characterized by high intake of animal proteins and saturated fats, not only increase circulating and urinary levels of the TMA-derived metabolite TMAO but also shift gut microbial composition [26]. Consistent with this, Manor et al. [27] reported that individuals consuming animal-based diets and those with symptomatic CVD exhibit higher gut abundance of *Desulfovibrio*, which was positively associated with elevated TMAO levels.

Beyond digestion and barrier protection, the gut also functions as an endocrine organ. Enteroendocrine cells release hormones in response to luminal and neuronal signals. These cells secrete serotonin (5-HT), which influences motility, secretion, and central nervous system function. They also secrete glucagon-like peptide 1 (GLP-1) and peptide YY (PYY), which regulate insulin secretion, satiety, and motility. Together, these gut-derived hormones coordinate digestion, appetite, and systemic metabolism, highlighting the important role of the gut microbiota in human physiology [28]. Dietary components that are not readily absorbed in the small intestine serve as substrates for the gut microbiota [29], which directly influences its composition and metabolic processes, potentially affecting TMAO metabolism [30]. Therefore, diet is a significant determinant of the structure, diversity, and functional activity of gut microbiota [31,32,33]. Previous studies have shown that a diet high in saturated fat increases the risk of metabolic disorders, including obesity and heart disease, through the microbiota [34,35,36]. The general trend in the literature suggests that diets high in saturated fatty acids are commonly associated with a characteristic high-fat diet (HFD) profile, marked by obesity. In contrast diets abundant in polyunsaturated fatty acids tend to exert beneficial effects [37]. To explore this topic further, this review specifically examined how various dietary fat types influence gut microbiota-derived TMAO.

## 2. Association of Dietary Habits with the Human Gut Microbiota

The relationship between the gut microbiota and diet is established early in life and persists throughout life. Early-life nutrition, especially breastfeeding, plays a crucial role in gut microbiota development [38]. Breastfeeding strongly correlates with increased *Bifidobacterium* abundance [39,40,41], a key component of healthy infant microbiota, along with *Lactobacillus* species [42]. Breast milk contains oligosaccharides, including lactose and more than 1000 distinct non-digestible molecules that serve as ideal substrates for bacterial fermentation. Preterm infants typically show altered microbial profiles dominated by Proteobacteria, but breast milk feeding specifically increases *Bifidobacterium* populations, indicating how the non-digestible sugars in breast milk selectively promote beneficial bacterial species. As infants transition to solid foods, their microbiota adapts to broader range of energy substrates, enhancing carbon metabolism capacity [43]. Because multiple factors, such as nutrition, surroundings, time of year, and personal well-being, influence it, defining a standard or typical microbiota profile for the general population is practically impossible [38]. Despite this variability, human microbiota can be categorized into three distinct enterotypes based on predominant genera: *Bacteroides*, *Prevotella*, and *Ruminococcus* [31], with Firmicutes and Bacteroidetes representing the most abundant phyla in human fecal samples [44]. These enterotypes are shaped mainly by long-term dietary patterns. Diets high in protein and animal faty are associated with increased levels of *Bacteroides*, whereas carbohydrate-focused diets are associated with increased levels of *Prevotella*. Interestingly, these enterotypes appear to be independent of external elements such as age, sex, body mass index, or place of residence and are primarily influenced by diet and genetics [45].

Recent studies suggest that enterotypes may lie on a spectrum rather than being distinct categories, but they remain useful tool for exploring overall microbiota diversity [46]. When participants were grouped into previously identified enterotypes based on their fecal microbial profiles, those with an enterotype dominated by the genus *Prevotella* (n = 4) exhibited significantly higher plasma TMAO levels than those with an enterotype marked by a higher abundance of the genus *Bacteroides* (n = 49) [3]. Although this finding supports a potential connection between enterotypes and microbial TMA generation, caution is warranted when interpreting or generalizing the results. Notably, only four samples in the study belonged to the *Prevotella* group, and the *Ruminococcus* enterotype was entirely absent [3].

## 3. High-Fat Diets Modify Gut Microbiota and Influence TMAO Production

HFDs alter the gut microbiota composition, notably increasing the proportion of Firmicutes relative to Bacteroidetes [47,48]. While this elevated ratio is often considered a marker of obesity in animals and humans, obese individuals generally show lower bacterial diversity compared to lean counterparts. This suggests that compositional changes at the family, genus, or species level may have a greater impact than the phylum-level ratio alone [49]. Thus, relying solely on the Firmicutes to Bacteroidetes ratio may oversimplify the complex relationship among microbial diversity, composition, and health outcomes. Instead of a single universally healthy microbiota state, multiple beneficial microbiota profiles may exist, each characterized by distinct microbial communities. For example, the relationship between Bacteroidetes abundance and microbiota diversity is non-linear in humans [50]. Beyond taxonomic shifts, microbiota composition is closely linked to host clinical markers and lifestyle factors, with specific host-microbe interactions playing a pivotal role. Gut microbiota contributes to various diet-induced metabolic disorders, including cardiovascular disease [1,11], obesity [51,52], insulin resistance [52,53], and non-alcoholic fatty liver disease [54,55,56]. HFDs increase cardiovascular disease risk partly through gut microbial production of TMA [19]. While gene clusters involved in choline metabolism are widespread among the gut microbiota [23], only facultative anaerobes tend to proliferate in individuals consuming HFDs [19]. By comparing the effects of high-fat and low-fat diets, researchers found that dietary choline (1%) provided *Escherichia coli* a CutC-dependent growth benefit in mice fed HFDs, whereas this effect was absent in mice fed a low-fat diet. This suggests that *E. coli* strains carrying the *cut* operon metabolize choline under conditions induced by HFD-mediated alterations in the intestinal environment [19]. In contrast to high-fat Western diets, the Mediterranean dietary pattern demonstrates multiple protective mechanisms against TMAO-exacerbated atherosclerosis and gut barrier dysfunction [57,58]. The Mediterranean diet is characterized by two key components that synergistically promote cardiovascular and gut health: *n*-3-rich fatty acids from regular fish consumption and abundant dietary fiber from plant-based foods, including vegetables, fruits, legumes, whole grains, and nuts. These fibers elevate fecal short-chain fatty acid concentrations as gut bacteria ferment host-indigestible carbohydrates. The resulting short-chain fatty acids strengthen the intestinal barrier and reduce systemic inflammation [57,59].

## 4. Do All Fatty Acids Have the Same Impact?

Fatty acids (FAs) serve as crucial energy sources, undergoing mitochondrial β-oxidation and catabolism through the tricarboxylic acid cycle. Beyond energy production, FAs are vital components of cell membrane phospholipids. The specific FA composition of cell membranes varies across cell types, membrane structures, and phospholipid classes, influenced by diet, metabolism, hormonal conditions, cell activation states, and genetics. This composition affects the physical properties of membranes, including fluidity and structural order, which in turn regulate the function and mobility of proteins within the membrane [60].

FAs exhibit significant diversity in their carbon chain lengths and degree of saturation [61]. By chain length, FAs are classified as short-chain (2−4 carbons), medium-chain (6−12 carbons), and long-chain (>12 carbons) fatty acids (SCFAs, MCFAs, and LCFAs, respectively) [62]. LCFAs, particularly C16 and C18, are the most abundant in mammalian cells. By saturation, FAs are classified as saturated (SFAs, no double bonds), monounsaturated (MUFAs, one double bond), or polyunsaturated (PUFAs, multiple double bonds). PUFAs are further divided into omega-3 (*n*-3) and omega-6 (*n*-6) families based on the position of the final double bond relative to the carboxyl end [61]. SCFAs (C4–C6) and MCFAs (C8–C12) can freely diffuse across the mitochondrial membranes, allowing direct accessing to the mitochondrial matrix. In contrast, LCFAs (C14–C20) cannot cross mitochondrial membranes unaided and require a carnitine-dependent transport system [63]. The transport of LCFAs into mitochondria involves a shuttle mechanism known as the carnitine cycle, which relies on three key enzymes: carnitine palmitoyltransferase I (CPT1), carnitine acylcarnitine translocase (CACT), and carnitine palmitoyltransferase II (CPT2). Although these enzymes are located in different regions of the mitochondria, all three enzymes require carnitine as a cofactor [63,64].

FA composition varies by dietary source [65]. Animal-derived fats are typically high in SFAs [66], while marine sources like fish oils are rich in *n*-3 PUFAs [67]. Vegetable oils display diverse FA profiles depending on plant species: olive oil is abundant in MUFAs, whereas sunflower and flaxseed oils are rich in PUFAs [68]. Palm oil contains 40–50% saturated fat, predominantly palmitic acid (16:0), making it significantly more saturated than olive or sunflower oils [69]. MCFAs comprise over 50% of the lipid content in coconut and palm kernel oils, but only 14–15% in cow milk, where levels vary with breed, pasture type, and season [70]. This diversity in FA composition reflects the unique characteristics and potential health impacts of each dietary source [68].

Common sources of dietary fats in HFDs, including plant oils (corn, peanut, soybean, sunflower) and animal fats (lard), generally increase Firmicutes and decrease Bacteroidetes, though their impact on gut microbiota alpha diversity varies. This variation may significantly influence health outcomes, as specific FA types can independently alter microbial composition [71]. Factors such as saturation level and carbon chain length appear to play key roles in shaping microbial communities [72]. Diets high in saturated FAs have been linked to reduced microbial diversity and richness, as well as decreased abundance of Bacteroidetes [73]. Mice fed lard-based HFD exhibit Firmicutes and reduced Bacteroidetes [73,74], a pattern also observed with palm oil-based HFDs [75]. In contrast, diets high in unsaturated FAs, such as fish oil, promote greater microbial diversity and richness while increasing Bacteroidetes [75]. Liu et al., (2012) [76] showed that SFAs more strongly altered gut microbiota profiles than *n*-3 or *n*-6 PUFAs, notably causing a significant Bacteroidetes reduction characteristic of obesity.

Although the exact mechanisms are not fully understood, *n*-3 FAs appear to support gut health by increasing lipopolysaccharide (LPS)-suppressing bacteria like *Bifidobacteria* and *Lactobacillus* while reducing inflammatory LPS-producing *Enterobacteria* [77,78,79]. Similarly, diets rich in MUFAs, such as those containing olive oil, are known to beneficially modify gut microbiota composition by expanding commensal bacterial populations [77,80]. Medium-chain triglycerides (MCTs) also protect against LPS-induced endotoxemia. Rats fed MCTs daily for one week survived subsequent intravenous LPS administration, while corn oil-fed rats did not, highlighting MCTs’ anti-inflammatory benefits [70].

The effect of dietary FAs on TMAO has been demonstrated in multiple studies [9,12,81,82,83,84,85,86,87,88,89,90,91], as summarized in Table 1. In animal studies, SFAs from palm oil (rich in palmitic acid, 16:0), lard (high in oleic acid, 18:1n-9, and palmitic acid, 16:0), butter, and egg yolk-derived phosphatidylcholine promote TMA-producing bacteria such as *E. coli* and *Desulfovibrio* [19,24,82,92,93]. MUFAs are generally associated with reduced TMAO levels [80,88], though this may reflect bioactive compounds in oil rather than the fatty acids themselves [81,94]. Andújar-Tenorio et al. (2022) [80] compared the effects of different HFDs in mice fed standard chow, butter-enriched chow (representing saturated fats), or extra-virgin olive oil (EVOO)-enriched chow (representing unsaturated fats). After 12 weeks, butter-fed mice exhibited higher *Desulfovibrio* abundance than EVOO-fed mice. EVOO’s protective effects may be partly attributed to its 3,3-dimethyl-1-butanol (DMB) content, a choline analog that inhibits microbial TMA lyases without bactericidal activity. In polymicrobial systems including intestinal contents and human feces, DMB reduces TMA production and lowers TMAO concentrations in mice consuming high-choline or carnitine diets [81].

*n*-3 PUFAs from fish oil, linseed oil, and *Symplectoteuthis oualaniensis*-derived phosphatidylcholine lower TMAO levels, increase SCFA-producing bacteria, and reduce *Desulfovibrionaceae* in animal studies [84,88,95]. However, *n*-3 PUFAs from krill oil, *n*-3-enriched eggs, and Mediterranean diet interventions showed neutral effects on TMAO in human clinical trials [85,86,96]. This animal–human discrepancy likely reflects greater variability in human baseline gut microbiota composition, genetic factors, and lifestyle habits that may mask FA-specific effects, whereas animal studies provide more controlled conditions [97,98]. *n*-6 PUFAs from sunflower oil and soybean phosphatidylcholine either increased TMAO or showed neutral effects in animal studies [89]. Transgenic mice expressing FAT-2 (converting MUFAs to *n*-6 PUFAs) exhibited higher choline and TMAO levels with increased *Enterobacteriaceae* and depleted *Bifidobacteriaceae* compared to wild-type and FAT-1 mice (converting *n*-6 to *n*-3 PUFAs), suggesting that *n*-6 PUFA effects on TMAO depend on the *n*-6/*n*-3 ratio [90]. SCFAs, particularly butyrate, inhibit microbial TMA production by downregulating TMA-lyase genes (CutC, CntA) and reducing TMA-producing bacteria (*Escherichia fergusonii* and *Anaerococcus hydrogenalis*) in vivo and in vitro models [9]. Mechanisms by which FAs influence gut microbiota and TMAO formation are outlined in Table 2.

**Table 1:** Summary of the effects of dietary fats and oils on TMAO and gut microbiota.

Fat/Oil/FA Source	Main FA Composition	Model	Main Findings on TMA-Producing Bacteria and TMAO	References
Palm oil	Palmitic acid (16:0) (SFA)	In vivo (animal)	Increased *E. coli* abundance, which is associated with higher TMAO production	[19,92]
Lard	Oleic acid (18:1*n*-9) (MUFA), Palmitic acid (16:0) (SFA)	In vivo (animal)	Promoted *Desulfovibrio* growth, a bacterium linked to elevated TMAO	[24,93]
Butter	Palmitic acid (16:0), Stearic acid (18:0) (SFA)	In vivo (animal)	Increased *Desulfovibrio* abundance compared to EVOO-fed mice	[24,80]
Extra-virgin olive oil	Oleic acid (18:1*n*-9) (MUFA)	In vivo (animal)	Reduced *Desulfovibrio* abundance; possibly due to DMB-mediated inhibition of microbial TMA lyases, leading to decreased TMA and TMAO production	[24,80]
Sodium butyrate supplement	Butyrate (C4:0) (SCFA)	In vitro; In vivo (animal)	Suppressed TMA formation by downregulating TMA-lyase genes (CutC, CntA), reduced *E. fergusonii* and *A. hydrogenalis*, lowered TMAO, and alleviated HFD-induced atherosclerosis	[9]
Sandalwood seed oil	Oleic acid (18:1n-9) (MUFA)	In vivo (animal)	Reduced plasma TMAO compared to sunflower oil (rich in linoleic acid, an n-6 PUFA)	[88,89]
Fish oil	EPA (20:5*n*-3), DHA (22:6*n*-3) (*n*-3 PUFAs)	In vivo (animal)	Lowered TMAO levels, increased SCFA-producing bacteria and *Bifidobacterium*, reduced *Desulfovibrionaceae*, and protected against TMAO-aggravated atherosclerosis	[83,88]
Linseed oil	α-Linolenic acid (18:3*n*-3) (*n*-3 PUFA)	In vivo (animal)	Reduced TMAO levels compared to sunflower oil and promoted SCFA-producing bacteria	[88,89]
Sunflower oil	Linoleic acid (18:2*n*-6) (*n*-6 PUFA)	In vivo (animal)	Increased TMAO levels compared to sandalwood seed oil, fish oil, and linseed oil	[88,89]
Phosphatidylcholine from *S. oualaniensis*	EPA, DHA (*n*-3 PUFAs)	In vivo (animal)	Prevented HFD-induced increase in serum TMAO	[84]
Phosphatidylcholine from soy-bean	Linoleic acid (18:2*n*-6) (*n*-6 PUFA)	In vivo (animal)	Showed neutral effects on TMAO compared with HFD control	[84]
Phosphatidylcholine from egg yolk	Palmitic acid (16:0), Stearic acid (18:0) (SFA)	In vivo (animal)	Elevated serum TMAO compared with HFD control	[84]
Chicken protein hydrolysate (CPH) ± chicken oil	Oleic acid (18:1*n*-9) (MUFA)	In vivo (animal)	Increased plasma TMAO and total carnitines in both CPH and CPH + chicken oil groups compared to control diet; unclear whether oleic acid alone drives this effect	[87]
Krill oil supplement	EPA:DHA (*n*-3 PUFAs enriched phospholipid)	In vivo (human, clinical)	Improved cardiovascular risk markers (TAGs, lipoproteins, FA profile, redox balance), but did not affect plasma TMAO or carnitine	[85]
*n*-3–enriched eggs	α-Linolenic acid (18:3*n*-3), EPA, DHA (*n*-3 PUFAs)	In vivo (human, clinical)	Increased plasma choline and betaine levels, but did not alter TMAO	[86]
Mediterranean diet	1:2:5 ratio of *n*-3 PUFA:SFA:MUFA	In vivo (human, clinical)	Found no significant changes in plasma TMAO or TMAO-to-precursor ratios in either the Mediterranean diet group or the Healthy Eating comparison group, despite both groups having elevated fasting levels at baseline	[96]
High- vs. low-SFA diets with protein variation	SFA; red meat, white meat, non-meat)	In vivo (human, clinical)	Revealed no effect of SFA content on TMAO, but red meat consumption increased TMAO via higher carnitine intake and reduced renal excretion	[12]
Western diet (WD)	SFA	In vivo (animal)	Showed a pronounced rise in plasma TMAO in mice fed a Western diet for 8 weeks compared with those on a standard diet	[91]

**Table 2:** Potential mechanisms by which different fatty acids influence gut microbiota and TMAO formation.

FA Class	FA Type	Potential Mechanism	Impact on TMAO
Saturated	Short-chain FAs	Direct Effects: Inhibiting *E. fergusonii* and *A. hydrogenalis*, key TMA-producing bacteria and downregulates TMA-lyase gene (CutC, CntA, and YeaW) expression, reducing TMAO formation [9]. Indirect Effects: Lowering colonic pH and promoting the growth of probiotics while exhibiting antibacterial activity against Gram-negative bacteria, including TMA producer *E. coli* [99,100,101]. Preventing gut inflammation [25,102], which promotes inflammatory nitric oxide production and may indirectly reduce TMA formation by inhibiting nitrate-induced *cut* gene expression [19].	Decrease
	Long-chain FAs	Direct Effects: Disrupting mitochondrial oxygen metabolism in enterocytes shifts gut conditions toward aerobiosis [19], favoring the expansion of facultative anaerobe *E. coli* [103], which increases TMA [19]. Increasing intestinal TMA-producing *Desulfovibrionaceae* [93] through elevated taurine-conjugated bile acids in the gut, thereby enhancing the availability of organic sulfur for sulfite-reducing microorganisms [93,104].	Increase
Unsaturated	Poly-unsaturated FAs (*n*-3)	Direct Effects: Reducing *Enterobacteriaceae* [105], a bacterial family linked to increased conversion of choline into TMAO precursors [19]. Indirect Effect: Enhancing SCFA-producing bacteria [25], potentially exerting the same effects as SCFAs. Increases LPS-suppressing *Bifidobacterium* [83], which negatively correlates with inflammation and plasma TMAO [106].	Decrease

## 5. Potential Mechanism

While most FAs are absorbed early in the digestive tract, excess amounts reach the colon, where they can alter the composition of the intestinal microbial community [107]. Unlike long-chain FAs (LCFA), short- and medium-chain FAs (SCFA and MCFA) are efficiently absorbed by intestinal epithelial cells through passive diffusion due to their shorter chain length and higher water solubility [70], minimizing colonic accumulation and microbial disruption.

Gut dysbiosis from HFDs occurs primarily through altered oxygen and energy availability [93,103,104]. The colon’s normally hypoxic environment favors beneficial anaerobes while limiting facultative anaerobes such as *Enterobacteriaceae* [108]. HFDs impair enterocyte mitochondrial function and oxygen consumption, increasing luminal oxygen and nitrate levels [19]. This shift favors facultative anaerobes such as *E. coli* while suppressing obligate anaerobic bacteria [19], elevating the Gram-negative to Gram-positive bacterial ratio [66]. Enhanced LPS release from Gram-negative bacteria stimulates inflammation, further promoting proinflammatory microbial populations. This dysbiotic profile specifically shows diminished obligate anaerobic Firmicutes and elevated facultative anaerobic *Enterobacteriaceae* levels [103]. Some commensal bacteria, like *E. coli*, exploit inflammatory conditions by using nitric oxides as an energy source, gaining a competitive advantage [109]. This *Enterobacteriaceae* expansion increases bacterial conversion of choline into TMA, the precursor to TMAO [19]. HFDs rich in saturated fat may also facilitate TMAO production by increasing the availability of organic sulfur to sulfate-reducing bacteria, such as *Desulfovibrionaceae*, including *Desulfovibrio* [93]. When dietary fat is consumed, the liver synthesizes bile acids [110], producing taurine- or glycine-conjugated bile acids that are released into the duodenum to facilitate lipid absorption in the small intestine. During intestinal transit, most bile acids undergo deconjugation [111,112], releasing taurine and thereby increasing sulfur availability for sulfate-reducing organisms [93,104]. Furthermore, hydrophobic bile acids compromise intestinal barrier integrity, potentially allowing microbial metabolites to enter the bloodstream [112], such as TMA.

Another mechanism by which FAs may alter gut microbiota composition is through their antimicrobial properties [99]. Their potency depends on chain length, degree of saturation, and the position of double bonds [107,113,114]. SFAs are generally more effective at shorter chain lengths, while MUFAs and PUFAs tend to exhibit greater activity at longer chain lengths. SCFAs display pH-dependent antimicrobial activity, disrupting energy metabolism primarily in Gram-negative bacteria [99]. Higher SCFA concentrations reduce pH, favoring *Bifidobacteriaceae* and *Lactobacillaceae* over *Enterobacteriaceae* [99,113]. SCFAs suppress pathogenic bacteria, preventing dysbiosis and inflammation [25,100,101], while reducing nitric oxide production that drives TMA lyase expression [19]. Colonic acidification also limits bile acid solubility, reducing colonocytes toxicity [113]. Notably, SCFA and TMAO production are inversely related, as HFDs elevate TMAO while suppressing SCFA synthesis [115]. MCFAs (particularly lauric acid) and n-3 PUFAs (EPA and DHA) display antimicrobial properties against Gram-positive bacteria by compromising cell membranes [70,114]. While these antimicrobial effects of SCFAs, MCFAs, and n-3 PUFAs may contribute to preventing gut dysbiosis and inflammation [25,100,101], and potentially reduce TMA lyase expression [20], direct experimental evidence directly linking fatty acid-induced antimicrobial effects to reduced TMAO levels is lacking. n-3 PUFAs also counteract dysbiosis through anti-inflammatory effects that enhance SCFA-producing microorganisms and inhibit LPS production [83,116,117], a key driver of gut inflammation [118]. Additionally, n-3 PUFA supplementation increases probiotic genera such as *Bifidobacterium* and *Lactobacillus* while reducing *Enterobacteriaceae* [105].

Dietary fats also shape gut microbiota through circadian regulation. Gut microbiota display diurnal oscillations that regulate the intestinal clock [119], with SCFAs fluctuating across the day and influencing clock gene expression (*PER2, PER3, ARNTL*). HFDs disrupt these rhythms by reducing oscillations in SCFA-producing taxa, altering circadian control of lipid metabolism [120]. Circadian disruption from light exposure and feeding patterns thus represents another pathway linking dietary fat–microbiota interactions contribute to metabolic disorders.

## 6. The Role of Gut Microbiota in Regulating Flavin Monooxygenase 3 Under HFD Conditions

Several studies demonstrate that high-fat diets (HFDs) increase hepatic flavin monooxygenase 3 (FMO3) expression through gut microbiota-dependent mechanisms [3,121]. Research in mice showed that HFD combined with oral carnitine significantly elevated FMO3 expression and plasma TMAO levels compared to HFD alone, while subcutaneous carnitine did not produce this effect indicating that gut microbial metabolism is essential for FMO3 upregulation [121]. A large clinical study (>2500 participants) confirmed that elevated plasma carnitine predicts increased cardiovascular events and mortality only in individuals with high TMAO levels, establishing TMAO as a critical pro-atherosclerotic factor [3].Further research confirmed the microbiota-dependent nature of FMO3 regulation by demonstrating that polyphenolic compounds (corilagin, gallocatechin gallate, epigallocatechin gallate) reduce FMO3 expression by binding microbial CutC protein and inhibiting TMA production [94]. These findings underscore the gut microbiota’s central role in FMO3/TMAO pathways and suggest that targeting microbial enzymes may offer therapeutic strategies for TMAO-related metabolic disorders.

## 7. Conclusions and Future Perspectives

HFDs significantly alter gut microbiota composition, often leading to metabolic disorders such as obesity, diabetes, non-alcoholic fatty liver disease, and cardiovascular disease. The degree of FA saturation and chain lengths appear to be key determinants of their gut microbiota-modulating effects. While saturated LCFAs have been shown to promote TMA-producing microbial populations, SCFAs and *n*-3 PUFAs reduce these microbes, indicating that both the type and quantity of dietary FAs significantly influence gut microbiota composition and related disorders. A clearer understanding of how dietary fatty acids affect gut microbiota and metabolic health requires future studies focusing on several key areas. First, more controlled experimental designs are needed where diet composition remains consistent except for the FA type being studied. This can be achieved through FA supplementation rather than using whole oils or fat sources that contain multiple bioactive components. For example, 3,3-dimethyl-1-butanol (DMB), a natural component of olive oil and structural analog of choline, inhibit TMA formation by targeting microbial TMA lyases, raising questions about MUFA findings when olive oil is used as the source. Moreover, certain oils contain polyphenols that independently influence gut microbiota, highlighting the need to control these additional variables. Second, research on the effects of MCFAs and their effects on TMAO levels remains limited despite their growing use in ketogenic diets and MCT-based nutrition. Third, dose–response relationships must be established through well-designed human trials to characterize the dose-dependent effects of different fatty acids on TMAO production and identify thresholds for beneficial or harmful effects. Addressing these gaps is essential for developing targeted dietary and therapeutic strategies to improve metabolic health.

## Data Availability

No new data were created or analyzed in this study. Data sharing is not applicable to this article.
